# Pitolisant and Other Histamine-3 Receptor Antagonists—An Update on Therapeutic Potentials and Clinical Prospects

**DOI:** 10.3390/medicines7090055

**Published:** 2020-09-01

**Authors:** Victoria Harwell, Pius S. Fasinu

**Affiliations:** Department of Pharmaceutical Sciences, College of Pharmacy and Health Sciences, Campbell University, Buies Creek, NC 27501, USA; vcharwell0609@email.campbell.edu

**Keywords:** cataplexy, excessive daytime sleep, histamine-3 receptor antagonists, narcolepsy, pitolisant, sleep

## Abstract

**Background:** Besides its well-known role as a peripheral chemical mediator of immune, vascular, and cellular responses, histamine plays major roles in the central nervous system, particularly in the mediation of arousal and cognition-enhancement. These central effects are mediated by the histamine-3 auto receptors, the modulation of which is thought to be beneficial for the treatment of disorders that impair cognition or manifest with excessive daytime sleepiness. **Methods:** A database search of PubMed, Google Scholar, and clinicaltrials.gov was performed in June 2020. Full-text articles were screened and reviewed to provide an update on pitolisant and other histamine-3 receptor antagonists. **Results:** A new class of drugs—histamine-3 receptor antagonists—has emerged with the approval of pitolisant for the treatment of narcolepsy with or without cataplexy. At the recommended dose, pitolisant is well tolerated and effective. It has also been evaluated for potential therapeutic benefit in Parkinson disease, epilepsy, attention deficit hyperactivity disorder, Alzheimer’s disease, and dementia. Limited studies have shown pitolisant to lack abuse potential which will be a major advantage over existing drug options for narcolepsy. Several histamine-3 receptor antagonists are currently in development for a variety of clinical indications. **Conclusions:** Although limited clinical studies have been conducted on this new class of drugs, the reviewed literature showed promising results for future additions to the clinical indications of pitolisant, and the expansion of the list of approved drugs in this class for a variety of indications.

## 1. Introduction

Histamine (2-(imidazol-4-yl) ethylamine), an endogenous monoamine, is one of the most studied chemical mediators in the biological systems involved in various physiological and pathological processes including cell proliferation and differentiation, tissues regeneration, wound healing, and immunomodulation [1,2,3]. First synthesized in 1907 and characterized in 1910, histamine and its roles in mediating anaphylactic reactions and body secretions have been explored for the development of a wide range of drugs referred to as antihistamines whose pharmacological effects are receptor-dependent. Earlier studies of the distribution and functions of histamine receptors led to the differentiation of histamine-1 (H1R) and histamine-2 (H2R) receptors in 1966 [4,5]. The histamine-3 receptor (H3R), discovered in 1983, was determined to be a presynaptic autoreceptor that inhibits histamine release in the brain [6]. A 445-amino-acids G-protein-coupled receptor, highly selective and specific for histamine, H3R has been described as a heteroreceptor for regulating the release of other neurotransmitters. Highly expressed in the basal ganglia, globus pallidus, hippocampus, and cortex of the CNS, the H3Rs play important roles in cognition compared to the H1R and H2R whose roles are mostly peripheral [7].

**Figure 1:**
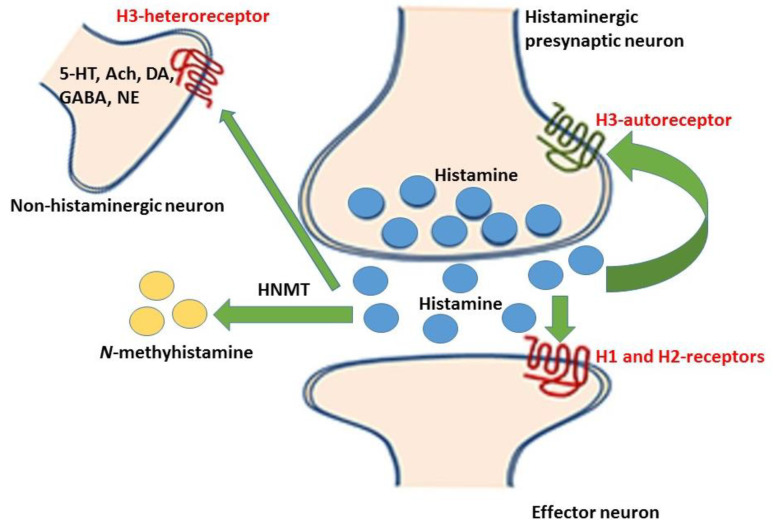
Released histamine (represented by the blue dots) from the histaminergic neurons bind to histamine-1 and -2 receptors on the effector neurons, while some are metabolized to the *N*-methylhistamine by the histamine *N*-methyltransferase (HNMT) enzyme. Some of the released histamine binds to the histamine-3 autoreceptors (on the histaminergic neurons) and the histamine-3 heteroreceptors (on the non-histaminergic neurons) thereby inhibiting the reuptake of released neurotransmitters. The central effect of this inhibition includes the enhancement of wakefulness, cognition, learning, and memory [13].

H3R activation is associated with arousal, cognition, fluid, food and temperature homeostasis, cardiovascular control, anxiety, and pain perception. When activated, H3R causes Gαi/o-protein-coupled inhibition of adenylate cyclase and the Na+/H+ exchange. It also stimulates the GTPγS binding, phospholipase A2 mitogen-activated protein kinase, and GSK-3β and AKT. The stimulation of the H3R receptor can impair cognition and many of the signaling pathways it is associated with are connected to long-term plasticity, neuronal cell death, and neuronal migration/neuroprotection [8]. The effect of H3R agonism includes the inhibition of neurotransmitter release with potential benefits in allergic rhinitis, cardiac dysfunction, and ventricular arrhythmias, all of which are triggered by excessive norepinephrine levels (Figure 1). Antagonism of the H3R enhances the release of the neurotransmitters histamine, acetylcholine, dopamine, norepinephrine, and others. An enhanced release of histamine can be beneficial in the therapeutic management of disease conditions associated with excessive daytime sleepiness (EDS) such as narcolepsy, parkinsonism, and obstructive sleep apnea (OSA). Enhanced histamine release is also known to enhance cognition, which is considered beneficial in such disease states as attention deficit hyperactivity disorder (ADHD), Alzheimer’s disease, and schizophrenia. The mechanism behind the potential benefit in ADHD includes the blockade of decreasing impulsivity, improving attention, and enhancing learning and memory [9]. The mechanism behind the potential therapeutic use in Alzheimer’s disease includes the reported increase of histamine in the frontal cortex and hippocampus but there are also reports of decreased histamine in the hippocampus and other areas of the brain. H3R antagonism is theorized to improve recognition memory, spatial memory, memory consolidation, and working memory [10,11,12].

The aim of the current review is to provide an update on H3R receptor antagonists including pitolisant, which have been approved for clinical use, and others in development.

## 2. Methods

A literature search was completed in June 2020 on the PubMed database, Google Scholar, and clinicaltrials.gov. There were no time restrictions implemented on when articles were published. The search strategy was constructed to find relevant articles addressing the therapeutic use of pitolisant and other H3R antagonists in humans. “Pitolisant” and “H3 antagonist” were the search terms used. Articles for extensive reviews included those that reported studies in human subjects and had full text availability. All titles and abstracts were independently screened. Studies included were limited to those published in English language. 

## 3. Results

**Figure 2:**
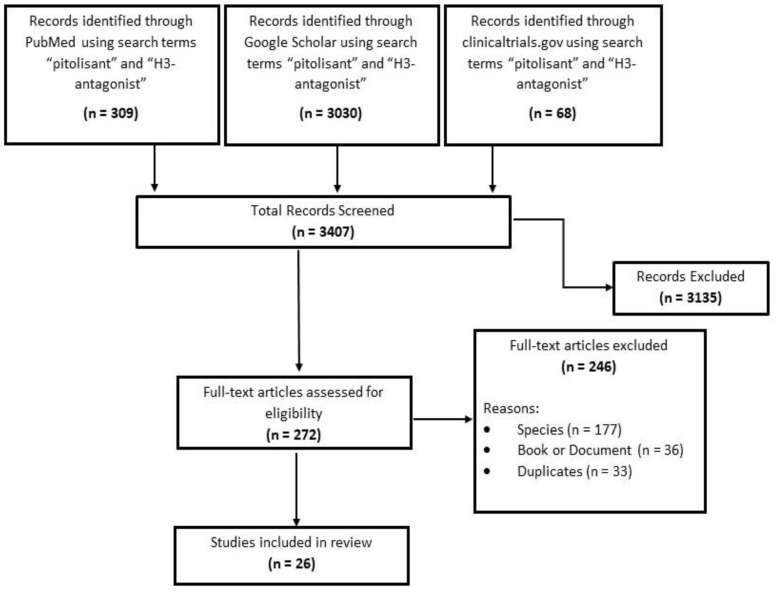
Search results and study selection.

H3R antagonists are a new class of drugs with limited experience in clinical utility. Only 26 publications qualified for inclusion for extensive review for this study. Figure 2 shows the chart for the search details.

### 3.1. Histamine-3 Receptor Antagonists as Therapeutic Agents

**Figure 3:**
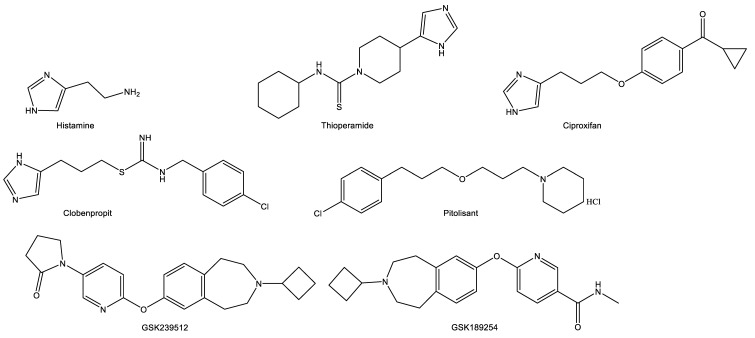

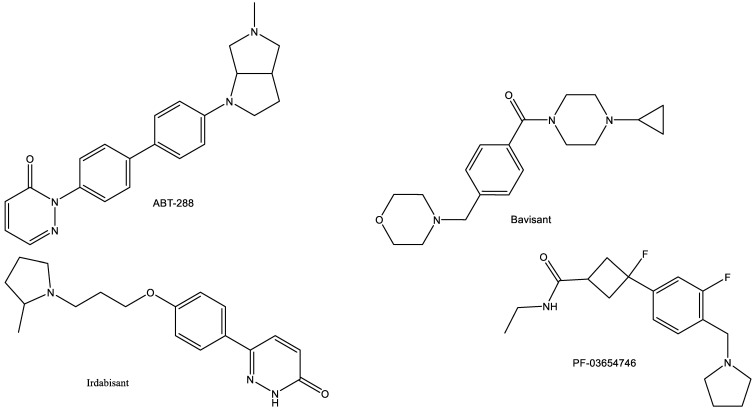
Structures of selected histamine-3 receptor antagonists in relation to histamine.

Since the discovery of the H3Rs and their rich expression in the CNS, researchers have continued to explore their therapeutic potential. The antagonism of H3Rs provides inverse agonism due to the inhibition of the feedback mechanism at high levels of the endogenous receptor ligands. The initial compounds developed for binding to the H3R were structurally related to histamine. This was largely achieved by retaining the imidazole ring with the substitution of, or modification to, the ethylamine side chain (Figure 3). For ionic interactions to occur in H3R antagonism, an aliphatic tertiary amine substituted by linking an alkyl chain was thought to be necessary [14]. Early results yielded several compounds including thioperamide (a potent H3R antagonist) and (R)-alphamethylhistamine (a potent H3R agonist) (Figure 3). However, the imidazole-based compounds screened displayed low H3 receptor selectivity and extensive hepatic metabolism which raises concern for cytochrome P450 (CYP)-mediated drug interactions. The replacement of the imidazole ring with nitrogen-containing heterocycles like pyrrolidine, piperidine, azepane, piperazine, diazepane, tetrahydrobenzo[d]azepine, and others appeared to overcome these challenges. For example, the removal of the imidazole ring resulted in compounds with increased H3R affinity, enhanced CNS penetration and reduced affinity for CYP interaction [15,16,17,18].

### 3.2. Pitolisant—Summary on the First Histamine-3 Receptor Antagonist Approved for Clinical Use

Pitolisant (Wakix™), is a first-in-class H3R antagonist/inverse agonist which received initial approval in the United States in 2019 for the treatment of EDS in patients with narcolepsy. Prior to its approval in the United States, pitolisant was approved in Europe for the treatment of narcolepsy with or without cataplexy. Before these approvals, it had orphan drug designation in the EU and the United States.

Pitolisant, 1-(3-(3-[4-chlorophenyl]propoxy)propyl)piperidine hydrochloride, binds to the H3R with high affinity and selectivity, with no appreciable binding to other histamine receptors. It is a piperidine ring-containing structure with an ether linkage to a chlorobenzene (Figure 3). In pitolisant, the imidazole ring from the original pharmacophore has been replaced with a piperidine ring. This decreased the strength of interaction with CYP.

Available as oral tablets (strengths of 4.45 mg and 17.8 mg in the United States, and 4.5 and 18 mg in Europe), the recommended daily dose of between 17.8 mg and 35.6 mg, titrated from lower doses over 2–3 weeks, has been shown to be effective for approved indications. However, the achievement of clinical response could take up to eight weeks. The most common adverse effects of pitolisant include insomnia, nausea, and anxiety. It may increase the QT interval, thus, concurrent intake with antiarrhythmics, antipsychotics, and other drugs that elongate the QT should be avoided. 

There is currently insufficient human data on the safety of pitolisant in pregnancy and breastfeeding. At the maximum clinically beneficial dose of 35.6 mg, steady state C*_max_* of 73 ng/mL and an area under the curve (AUC) value of 812 ng*hr/mL was reported. Steady state was reached by day 7 and increased proportionally with the dose. With no significant food effect, 90% bioavailability is achieved, and the maximal absorption time is about 3.5 h. Pitolisant is well distributed into the tissues, with a volume of distribution of about 700 L (5–10 L/kg) and 91%–96% serum protein binding. The majority of administered pitolisant is metabolized largely by CYP2D6 and to a lesser extent, CYP3A4, to inactive metabolites. Renal clearance account for <2% of total clearance. 

Several factors that can influence the pharmacokinetic parameters of pitolisant is summarized in Table 1. 

### 3.3. Clinical Development of Pitolisant for Narcolepsy with or without Cataplexy

Pitolisant is currently the only H3R antagonist/inverse agonist approved for the treatment of narcolepsy with or without cataplexy in the United States and Europe. Narcolepsy is a chronic neurologic disorder of hypersomnolence which onset occurs in adults. It is characterized by EDS, the presence of which is required for diagnosis [19]. Cataplexy is an abrupt and reversible loss of muscle tone during wakefulness, usually triggered by a strongly emotional stimulus [20]. Cataplexy occurs in about 60% of narcolepsy patients [21]. Narcolepsy is a consequence of the selective loss of hypocretin-producing neurons in the hypothalamus. Hypocretin signaling is responsible for the synthesis of the wake-promoting excitatory neurotransmitters including histamine, serotonin, norepinephrine, and dopamine [22]. While the pathophysiology of cataplexy is not totally clear, it is well understood that the hypocretin-producing neurons stimulate the areas of the brain that are responsible for the rapid-eye movement (REM) sleep whether due to functional interactions or other mechanisms [23]. Substantial loss of these neurons may disrupt REM sleep with consequent cataplexy attacks [24]. The aim of treatment for narcolepsy is to improve wakefulness and reduce cataplexy attacks.

In the early stage of its discovery, pitolisant, even at high concentrations, demonstrated selectivity for the H3R in the presence of several other receptors [25]. It induced central histaminergic and adrenergic transmissions in animal models, increasing wakefulness and decreasing episodes of REM sleep. In a single-blind crossover pilot study in twenty-two patients with narcolepsy who were given placebo for one week followed by pitolisant 40 mg daily for another week, the Epworth sleepiness scale (ESS) showed a baseline reduction from 17.6 by 5.9 by pitolisant compared to only 1.0 with the placebo; with attendant significantly reduced EDS on the last days of pitolisant dosing [26].

While several clinical studies have demonstrated the safety and efficacy of pitolisant (Table 2), three pivotal clinical trials including the Harmony I, Harmony III, and Harmony-CTP led to the final regulatory approval of pitolisant for the treatment of narcolepsy. 

Harmony I was a double-blind, randomized trial that compared pitolisant with placebo or modafinil in patients with narcolepsy [27]. Included in the study were adult participants with a diagnosis of narcolepsy confirmed by polysomnogram and a multiple sleep latency test (MSLT) within the preceding 5 years, showing a mean sleep latency of ≤8 min, sleep onset REM periods of ≥2 min, in addition to an ESS score of ≥14 (out of 24). The study randomized a total of 95 patients with 94 (31 pitolisant, 30 placebo, and 33 modafinil) included in the intention-to-treat analysis. The treatment included a 3-week flexible (dose titration of pitolisant (10–40 mg) and modafinil (100–400 mg)) and 5-week stable dosing periods. The primary endpoint was to assess the difference in change in ESS scores between the groups. By week 8, all groups showed significant improvements from baseline on ESS and maintenance of wakefulness test (MWT) sleep latency. Response rate, based on the final ESS scores, was 45% (pitolisant), 46% (modafinil), and 13% (placebo) for the groups, showing a pitolisant treatment effect of 4.4 (95% CI: 2.1–9.2; *p* < 0.0006) compared to placebo; and 1.0 (95% CI: 0.68–1.6; *p* = 0.908) compared to modafinil. Pitolisant was superior to placebo (with a mean ESS decrease of 5.8) but did not demonstrate non-inferiority to modafinil. Performance on the secondary efficacy endpoints included 81%, 86%, and 56% of participants on pitolisant, modafinil, and placebo, respectively, who showed improvement on the Patient Global Impression of Change (PGIC); and 73%, 86%, an 56% on Clinician Global Impression of Change (CGI-C) scale. There was significantly reduced incidence of reported cataplexic attacks with pitolisant compared to placebo, although similar to modafinil.

HARMONY Ibis, an 8-week multi-center phase III clinical trial, was similar to HARMONY I in design and objectives and endpoints. It lasted for 8-weeks after an initial 2-week washout/withdrawal from psychostimulants by participants. A total of 166 patients were randomized (pitolisant 67, modafinil 66, and placebo 33) where the pitolisant group was treated with much lower dose range (5–20 mg/d) than in HARMONY I [28]. Although, the study analysis after 8 weeks showed that pitolisant caused significantly greater change in MWT sleep latency than the placebo (but similar to modafinil), the ESS score changes from the baseline did not demonstrate pitolisant to be superior. Pitolisant did not significantly reduce the rate of cataplexy attacks.

The long-term safety and efficacy of pitolisant (titrated up to 40 mg/day) for the treatment of narcolepsy was assessed in HARMONY III clinical trial [29]. Participants included patients with narcolepsy with/without cataplexy, and persistent EDS (ESS ≥ 12) (despite established treatments). Unlike in HARMONY I, participants in HARMONY III could have concurrent CNS stimulants and anticataleptic medication throughout the trial, could be previously pitolisant-exposed, or pitolisant-naïve patients. Participants were titrated in the first month of the trial to a stable pitolisant dose. The primary endpoint was the incidence of treatment emergent adverse effects (TEAE) at 12 months. Secondary efficacy endpoints included changes in ESS score, among others. A total of 102 patients including 29 previously pitolisant-exposed (23 with cataplexy), and 79 pitolisant-naïve (52 with cataplexy) patients were included in the 12-month trial. Of the total enrolment, 35% were taking other medications including stimulants and antidepressants, for narcolepsy. Only 68 patients completed ≥12 months of treatment, with 31 of the total withdrawals happening in the first 3 months of study. Withdrawals due to adverse effects were 9 in the pitolisant-naïve group, and 2 in pitolisant-exposed group. Most withdrawal (*n* = 20) was due to perceived insufficient efficacy.

At baseline, patients in the exposed treatment group had lower ESS and European QOL scores. Patient’s taking additional anti-narcolepsy medications showed a significant increase in TEAE’s as well as treatment related TEAE’s. There was no significant difference in severe and serious TEAE’s between treatment-naïve and treatment-exposed patients. TEAE’s leading to withdrawal was not different between groups. Out of the 102 patients studied, 11 had a severe TEAE considered to be related to the study drug. This included migraine (2), insomnia (1), irregular sleep (1), premature ejaculation (1), and spontaneous abortion (1). Compared to baseline, mean ESS scores decreased along the 12 months period with the maximum effect being shown at 6 months.

Among the participants who completed 12 months of treatment, there was a mean 4.6 change in ESS scores from baseline to end of study (with most decrease occurring in treatment-naïve patients using pitolisant as monotherapy (mean ESS score change of 6.5)). All decreases between subtypes were significant except for treatment naïve patient’s taking concomitant sodium oxybate (mean ESS score change of 2). Among study completers with cataplexy records, there was a 68% mean decrease in cataplexy attacks. This trial also showed significant improvement in hallucinations and sleep paralysis. These results are consistent with results found in Harmony I but showed long-term safety and sustained effect after 1 year. This trial did not include a placebo but had improved external validity when compared to Harmony I.

Harmony-CTP, a 7-week double-blind placebo-controlled phase IIII trial focused on the safety and efficacy of pitolisant who had narcolepsy with cataplexy [30]. To be included, patients needed to be diagnosed with narcolepsy with cataplexy according to the International Classification of Sleep Disorders 2 (ICSD-2) criteria, have an ESS score of ≥12 at baseline as well as three or more cataplexies per week. Ongoing anticataleptic treatment sodium oxybate or antidepressants were allowed as long as the patient has been on a stable dose 1 month prior to randomization. Participants were randomized into pitolisant (*n* = 54) and placebo (*n* = 52) groups after the initial 2-week period of screening. The first 3 weeks consisted of dose-titration (5–20 mg/day) to stability, followed by a 4-week period of stable dosing (5–40 mg/day). Patients taking placebo were twice as likely to be taking concomitant cataplexy medications throughout the trial.

The primary outcome of this study was the change in average number of cataplexy attacks per week between the baseline period and the 4-week stable dosing period. The reduction of cataplexy by the pitolisant group was 75%, and this remained consistent with stable doses of 10, 20, and 40 mg. There was no statistical difference in effect between monotherapy and patient’s taking concomitant medications. The significance in cataplexy rates began at week 3 of treatment (*p* < 0.0079).

Superiority was observed with pitolisant in all secondary endpoints including reduced weekly cataplexy rate (WCR), mean change in ESS, MWT, clinical global impression of change, patient global opinion of efficacy, European QOL questionnaire, and number of days without hallucinations.

### 3.4. Potential Additional Indications for Pitolisant

The wake-promoting and cognition-enhancing effect of H3R antagonism is thought to hold potential therapeutic benefits in other CNS disorders. Pitolisant is being evaluated for some of these conditions.

#### 3.4.1. Epilepsy

Low levels of histamine have been associated with seizures. Thus, the enhancement of central histamine release by H3R antagonists has been thought as a potential pathway for the development of novel antiepileptic drugs [31,32]. Several synthetic H3R antagonists showed promising antiepileptic results in animal models [33]. In a multicenter, single-blind, placebo-controlled study in 14 participants, pitolisant suppressed epileptic discharges [34]. Pitolisant was given in 20, 40, and 60 mg doses with a matching placebo where the strongest efficacy and longest duration of effect were observed with the highest dose. In this photosensitivity proof of concept model, investigators observed for generalized epileptiform discharges on the EEG.

However, the efficacy of pitolisant in the treatment of epilepsy was not clearly evident in a French, exploratory multi-center study conducted in patients with refractory partial seizures [35]. Enrolled patients have had clinical diagnosis of epilepsy for at least 1 year, at least four partial-onset seizures per month during the 8-week baseline period, and a history of treatment failure with at least three appropriate antiepileptic drugs. While participants continued their antiepileptic drugs at the time, pitolisant, titrated (on individually determined basis) from 20 mg to a maximum of 40 mg per day, was given as adjunct treatment, over the 3-month study period. One-third of the 21 total patients had a reduction of at least 50% after 3 months of treatment with pitolisant, but the other two-thirds of patients had increased or maintained the number of seizures per week at the end of treatment. There were no seizure free patients in this population. It is important to note the limitations of this study including the small sample size, non-blinding, and the absence of placebo control. While further studies might be beneficial to draw a more conclusive inference, there has been no newer clinical studies on the efficacy of pitolisant in epilepsy treatment.

#### 3.4.2. Obstructive Sleep Apnea

Obstructive sleep apnea (OSA), often associated with cardiovascular, metabolic and/respiratory disorders, has very limited treatment options. In addition to a long-term effect of impaired attention, cognitive deficit, and reduced productivity and quality of life, patients with OSA commonly have complaints of fatigue and EDS. Current first-line treatment of OSA consists of continuous positive airway pressure (CPAP) which has a poor record of acceptance and adherence among patients [36,37].

Only one clinical study assessing the efficacy of pitolisant in OSA has been published [38]. In the phase III, double-blind, placebo-controlled, parallel-group, multicenter trial, 268 patients with moderate to severe obstructive sleep apnea, who had refused treatment with CPAP were randomized and given placebo or pitolisant. The 12-week trial began treatment with 5 mg, titrated for 2 weeks (up to 20 mg) based on individual response of efficacy and tolerance. Stable dosing was administered for 10 weeks thereafter. The per-protocol analysis included 181 patients in the pitolisant group and 61 in the placebo group. There were significant improvements in wakefulness in the pitolisant group compared to placebo. This included the reduction in the number and duration of sleep as well as sleepiness episodes showing significance in favor of pitolisant. Overall, Pitolisant reduced self-reported excessive daytime sleepiness as well as decreased ESS score showing clinical and statistical benefit in patients with OSA who refuse treatment with CPAP. While the potential benefit of pitolisant in OSA is much evident, the clinical merit of its use is not. One of the major concerns is the multisystem nature of OSA, and while sleepiness may be one of its prominent consequences, the underlying disease is not the target of pitolisant [39].

#### 3.4.3. Parkinson’s Disease

Daytime REM sleep dysregulation and narcolepsy-like symptoms are common in patients with Parkinson’s disease, disrupting wakefulness and reducing productivity [40]. Hypocretin cell loss is the underlying cause of EDS in Parkinson disease, as in narcolepsy [41]. Thus, pitolisant may be beneficial is Parkinson disease as an adjunct treatment. In a published article, Liguori and co-workers reported two cases regarding the use of pitolisant in patients with narcolepsy comorbid with Parkinson’s disease [42].

The first patient, a 68-year old with Parkinson disease and who was on standard treatment, had EDS occasioned by daily sleep attacks. After the diagnosis of narcolepsy comorbid with Parkinson disease was confirmed, therapy was initiated with pitolisant, titrated to 18 mg/day. There was improvement in EDS after 3 months with no sleep attacks reported, with further improvements reported at 6 months as measured by the ESS score. Pitolisant had no effect on the concurrent treatment for Parkinson disease.

In a second and similar patient, pitolisant improved EDS progressively after 3 and 6 months. However, sleep attacks, although reduced in frequency, persisted. The potential benefit of pitolisant in Parkinson disease will require further clinical investigations.

### 3.5. Pitolisant in Children

Current approval of pitolisant excludes use in pediatric population. For many patients diagnosed with narcolepsy, symptoms manifest before adulthood. Narcolepsy and daytime sleepiness can have a dramatic impact on children’s ability to perform physically, mentally, and academically. Having shown effectiveness in the adult population, pitolisant may be safe and effective in children with narcolepsy too. The need for newer anti-narcolepsy drugs is the more important considering that current treatment measured in children involve off-label drug use, with potentially serious side effects. Only one clinical stud has evaluated the use of pitolisant in children. In the open-label trial completed to evaluate the use of pitolisant as an alternative stimulant for narcolepsy-cataplexy in teenagers with refractory sleepiness, four teenagers were titrated 10–40 mg pitolisant [43]. The patients had tried available stimulants alone or in combination and were discontinued due to adverse effects or lack of efficacy.

All stimulants were stopped at least 3 days prior to the initiation of pitolisant. One patient continued a stable dose of escitalopram for cataplexy. The benefit of pitolisant was assessed by using an adapted ESS, polysomnographic parameters, and MWT along with subjective information from the patients. Three patients ended the trial on the maximum provided dose of 40 mg and one patient ended on 30 mg due to an adverse effect. At baseline, the mean adapted ESS score was 14.3, which decreased significantly to 9.3 with pitolisant monotherapy. The trial also allowed participants to begin other stimulant medications if efficacy was not being reached. Pitolisant was combined with methylphenidate, mazindol, and modafinil/sodium oxybate combination in three patients, respectively, while the fourth patient reached efficacy with pitolisant monotherapy. The addition of the adjunctive stimulants led to a further decrease of the adjusted ESS score by 7. Pitolisant improved daytime sleepiness in all patents, reduced cataplexy attacks in three patients. Reported adverse effects were similar to those known in adults. In addition to the limited sample size, the study was not placebo-controlled. As a trial conducted in teenagers, the relevance of its findings remains uncertain in younger children.

In two different publications, Pullen and co-workers reported the safe use of pitolisant in to treat EDS in children with Prader–Willi Syndrome [44,45]. The first study reported on ten children (2–16 years of age) dosed with pitolisant (4.5 mg/day, titrated up to 31 mg/day, based in individual basis, for up to 2 years in some participants) while the second was a case series of three children (ages 10, 12, and 15 years) who received 4.5–31.5 mg/day pitolisant for 14–16 months. In addition to decreased daytime sleepiness and improved cognition, pitolisant was shown to be safe in all participants.

Most recently, the tolerability of pitolisant was evaluated in a multi-center single-dose pharmacokinetic study in 25 children (6–17 years of age) with narcolepsy [46]. Apart from a significantly higher plasma exposure in younger vs. older children, pitolisant, at 17.8 mg, was well tolerated by all participants with only minor adverse events—headache, dizziness, diarrhea and vomiting-reported in a few. These limited studies suggest that pitolisant may be safe in children. With the availability of more safety and efficacy data in the future, the indications of pitolisant is expected to be broadened to include use in children.

### 3.6. Pitolisant Abuse Potential

All medications currently approved for the treatment of daytime sleepiness associated with narcolepsy are controlled substances with abuse potential. At the preclinical stage of development, while H3R stimulation enhanced central histaminergic and cholinergic transmission, dopamine turnover in the nucleus accumbens appeared unaffected [47]. This observation provided the first indication that pitolisant may be free of dependence liability.

In a single-dose, randomized, double-blind, active-and placebo-controlled, crossover study conducted to evaluate the human abuse potential of pitolisant in 38 volunteers, pitolisant demonstrated no potential for abuse [48]. The four-period crossover study compared therapeutic (35.6 mg) and supratherapeutic (213.6 mg) doses of pitolisant to phentermine (60 mg) and placebo in nondependent recreational stimulant users. Using a 100-point bipolar drug liking visual analog scale (VAS) (with 0 being dislike and 100 being strong like), the mean Emax scores were similar between pitolisant (both doses) and placebo, but significantly lower than those observed with phentermine. The result is similar with the secondary endpoints of “overall drug liking and willingness to take the drug again” with significantly higher mean Emax scores for phentermine compared to pitolisant (both doses), but with similar values for pitolisant and pitolisant. Overall, pitolisant, even at supratherapeutic doses, was shown to have no difference than placebo when it comes to abuse potential or safety.

### 3.7. Other Histamine-3 Receptor Antagonists in Development

Several other H3R antagonists are in different stages of development as potential therapies for EDS, schizophrenia, Alzheimer’s disease, ADHD, neuropathic pain, and allergies as shown in Table 3.

## 4. Discussion

The discovery of the H3R and its drug modulators represent a major medical advancement in recent history. The inhibition of the H3 autoreceptors enhances the release of wake-promoting and cognition-enhancing neurotransmitters including histamine and norepinephrine. This effect is beneficial in disease states such as narcolepsy, cataplexy, Parkinson disease, ADHD, Alzheimer’s disease, and dementia. The subsequent approval of pitolisant highlights the therapeutic success of H3R modulation. Pitolisant, being the first to be approved in the class, has been well demonstrated to be safe and efficacious in the treatment of narcolepsy with or without cataplexy. Meta-analysis of the clinical studies has shown that pitolisant is non-inferior to modafinil against EDS and superior for cataplexy treatment [60]. This is significant especially when combined with the probable freedom of pitolisant from abuse potential. Based on these and the promising results from other studies, there is a possibility of pitolisant becoming the first-line therapy for narcolepsy, other EDS-associated disorders, and as cognitive enhancing therapy in ADDH. This, most especially, in patients who have history of substance abuse.

Preclinical studies also suggest that the activation of the H3R may be beneficial in cardiovascular diseases. The exclusion of patients with cardiovascular disorders in the clinical trials that led to the initial approval of pitolisant limits the external validity of these studies. It also removes the potential evaluation of benefit or harm in this patient population. Since pitolisant is relatively new, experience from clinical utility is limited. Many of the studies reviewed, besides the standard clinical trials, are exploratory in nature.

Current evidence to support the use of pitolisant in other CNS disorders is sparse. While pitolisant may be potentially beneficial in Parkinson’s disease as shown in the two case reports evaluated, an actual controlled study is necessary for confirmation. Results from the limited study on the potential use of pitolisant in epilepsy is mixed. At the minimum however, pitolisant is expected to benefit epileptic patients who often experience drowsiness and sedation from their antiepileptic medications.

This review has sought to provide an update on pitolisant and other H3R antagonists. Apart from the limited amount of literature on this new class of drugs, the sample size of most of the reviewed studies present a major challenge, limiting external validation. The small sample size limits the ability to extrapolate and generalize the findings. Most of the studies were conducted in Europe and it will be important to evaluate pitolisant in other racial groups. Further studies hope to expand both the list and the clinical indications of H3R antagonists.

## 5. Conclusions

Since the discovery of HR3, several studies have explored the clinical significance of its modulation with drugs. Pitolisant is the first-in-class H3R antagonist to be approved for use in the treatment of narcolepsy with or without narcolepsy. At the recommended once-a-day dose of ≤36 mg, pitolisant is well tolerated and effective for its approved indications. Apart from adding to the available options for treating narcolepsy, pitolisant offers the advantage of lacking significant potential for abuse. Future additional indications related to EDS-associated disorders are expected based on promising early results. Several other H3R antagonists are in development for various clinical indications.

## Figures and Tables

**Table 1:** Effect of variable factors on the pharmacokinetic parameters of pitolisant.

Factor	Effect
Hepatic impairment	Increased total systemic exposure to pitolisant. In moderate hepatic impairment (Child-Pugh Class B), 17.8 mg maximum daily dose is allowed. In severe hepatic impairment, pitolisant is contraindicated.
Renal impairment	Increased maximum concentration and total systemic exposure to pitolisant. In moderate-severe renal impairment: 17.8 mg maximum daily dose is recommended. In end-stage renal disease, use is not recommended
Concomitant medications	Strong Inhibitors of CYP2D6 will increase maximum concentration and total systemic exposure. With such inhibitors, total daily pitolisant dose should not exceed 17.8 mg. CYP3A4 Inducers will decreases maximum concentration and total systemic exposure, maximum dose of 35.6 mg should be used. With CYP3A4 Inhibitors, no dose adjustment is necessary. No significant clinical difference when used alone or in combination with other common narcolepsy medications—modafinil and sodium oxybate.
Concomitant medications: pitolisant effect on other medications	Sensitive CYP3A4 substrates: Pitolisant decreased maximum concentrations and total systemic exposure. Sensitive CYP2D6 substrates: Slight increase in maximum concentrations and total systemic exposure. Other common narcolepsy medications: No significant difference when compared alone.
CYP2D6 Poor Metabolizers	Maximum daily dose should not excess 17.8 mg.
Food (fed vs. unfed state)	No significant effect.

**Table 2:** Relevant studies in the clinical development of pitolisant.

Trial Objective	Design	Outcome	Reference
To investigate the effectiveness of pitolisant in enhancing wakefulness in patients with narcolepsy	A pilot, prospective, comparative, sequential placebo-controlled, single-blind, multi-center study -Placebo given to 22 patients, daily for 1 week, followed by 40 mg of pitolisant daily for 1 week	Suppression to EDS by pitolisant. EDS not affected by placebo	[26]
To evaluate the efficacy of pitolisant in the treatment of patients with narcolepsy	HARMONY 1—a double-blind, randomized phase III clinical trial, comparing pitolisant vs. placebo or modafinil in patients with narcolepsy -Placebo: *n* = 30 -Modafinil 100–400 mg/day: *n* = 33 -Pitolisant 10–40 mg/day: *n* = 31 -8-week treatment period	Pitolisant at doses up to 40 mg was efficacious in the treatment of EDS compared to compared with placebo; it was well tolerated compared with modafinil -Pitolisant was superior to placebo (*p* = 0.024) -Pitolisant not non-inferior to modafinil (*p* = 0.250)	[27]
The assess the safety and efficacy pitolisant on cataplexy in patients with narcolepsy.	HARMONY CTP—a randomized, double-blind, placebo-controlled, parallel group Phase III trial -Pitolisant 5–40 mg/day: *n* = 54 -Placebo: *n* = 52 -7-week treatment period	Pitolisant was well tolerated and showed efficacy against cataplexy. Change in weekly cataplexy episodes between baseline and 4-week stable dosing period was significant beginning at week 3 of the study (*p* < 0.0079) and did not change based on dose	[30]
To evaluate the long-term safety and efficacy of pitolisant in patients with narcolepsy	HARMONY III -open-label, single-arm, multi-center (multi-country) study -Pitolisant 5–40 mg/day: *n* = 102 -1-year treatment period	The study confirmed long-term safety and efficacy of pitolisant on daytime sleepiness, cataplexy, hallucinations, and sleep paralysis. -The incidence of severe and serious TEAE’s at 12 months was not significantly different between placebo and pitolisant -12-month mean ESS decrease = 4.6	[29]
To assess the dose-dependent effects of pitolisant in the human photosensitivity model of epilepsy.	A multi-center, single-blind, placebo-controlled study -Single dose administration -Pitolisant 10, 20, 40, and 60 mg doses with matching placebo -*n* = 14	9 patients showed a statistically significant reduction in standardized photosensitive response. 60 mg doses were associated with insomnia and cognitive slowing in 2 patients	[34]
To assess the safety and efficacy of pitolisant as an adjunct treatment of refractory epilepsy	An exploratory multi-center, noncomparative, open-label Phase II trial -*n* = 23 -3-month treatment period	No conclusive evidence of efficacy. One-third of patients had a reduction of at least 50% in the number of seizures after 3 months of treatment, while seizure increased in another third of the participants.	[35]
To assess the safety and efficacy of pitolisant in the treatment of EDS in patients with OSA who refuse continuous positive airway pressure treatment	A double-blind, placebo-controlled, parallel-group, multi-center Phase III trial -12-week treatment period -Placebo: *n* = 60 -Pitolisant 5, 10, and 20 mg: *n* = 180	Pitolisant caused significant reduction in self-reported daytime sleepiness. It improved patient-reported and physician-assessed outcomes on severity of OSA -Statistically significant change in ESS from baseline and compared to placebo	[38]
To report clinical cases of pitolisant use in the treatment of narcolepsy comorbid with Parkinson’s disease.	Case reports -2 patients: 1 patient = 18 mg/day; 1 patient = 36 mg/day -6-month treatment duration	-Patient 1 had improvement in EDS with decreased ESS score from 17 to 10. The patient stopped experiencing sleep attacks. MOCA score was unaffected -Patient 2 had decreased ESS score from 19 to 9, sleep attacked decreased, but occurred occasional. MOCA score increased to 27 from a baseline of 22	[42]
To assess the safety and effectiveness of pitolisant in in patients with narcolepsy/cataplexy with severe daytime sleepiness, refractory to available treatments	-Off-label use of pitolisant -Open label -4 patients 16.5–18 years old -Pitolisant 10–40 mg/day	-Mean ESS decrease from 14.3 to 9.3 on monotherapy with pitolisant -When combined with other stimulants, mean ESS decreased to 7 -Cataplexy improved in 2 out of the 4 patients	[43]
To evaluate the abuse potential of pitolisant in adult patients with narcolepsy with or without cataplexy	-Single-dose, randomized, double-blind, active-and placebo-controlled, crossover study -Phentermine 60 mg -Placebo -Pitolisant 35.6 mg -Pitolisant 213.6 mg -38 study participants	-Mean Drug Liking Emax was significantly greater for phentermine compared to both doses of pitolisant (*p* < 0.0001) -Drug Liking Emax was similar for placebo and both doses of pitolisant	[48]

**Table 3:** Investigational histamine-3 receptor antagonists currently in various stages of clinical development.

Investigational Drug	Medicinal Chemistry	Target Indications	References
GSK239512	Potent, selective, orally bioavailable and brain penetrating H3RA. The imidazole ring is replaced with an azepane to increase H3R affinity (Figure 2).	Schizophrenia and mild-to-moderate Alzheimer’s disease and schizophrenia	[49,50]
ABT-288	The imidazole ring is replaced with a pyrrolidine	Schizophrenia and mild-to-moderate Alzheimer’s disease	[51]
Irdabisant (CEP-26401),	Pyrrolidine replaces the imidazole ring.	Cognition enhancement	[52]
Bavisant (JNJ-31001074)	Highly-selective, orally active, H3RA	ADHD and EDS in patients with Parkinson’s disease	[53,54]
GSK189254	imidazole ring is replaced with tetrahydrobenzo[d]azepine	Neuropathic pain	[55]
GSK1004723	Intranasal H1/H3 antagonist	Allergic and seasonal rhinitis	[56,57]
GSK835726	Oral H1/H3 dual antagonist	Allergic and seasonal rhinitis	[58]
PF-03654746	H3RA, imidazole ring replaced with a pyrrolidine ring	Allergic rhinitis, add on treatment for cognitive deficits in schizophrenia	[59,60]
SAR110894	H3RA	add-on therapy for mild-to-moderate Alzheimer’s disease	[61]
